# Winter-only grazing governs plant leaf economic traits and productivity pathways, with fertilization effects contingent on grazing regime

**DOI:** 10.3389/fpls.2026.1777082

**Published:** 2026-03-25

**Authors:** Minyue Liu, Hanyu Zhao, Kazhuo Cairang, Xuezhong Qi, Shuxin Li, Qiang Zhang, Yuliang Liu, Honglin Li

**Affiliations:** 1 State Key Laboratory of Plateau Ecology and Agriculture, Qinghai University, Xining, China; 2 College of Ecological and Environmental Engineering, Qinghai University, Xining, China; 3 Grassland Station in Henan Mongolis Autonomous County, Huangnan, Qinghai, China; 4 Dingning Town People's Government, Wuwei, China

**Keywords:** alpine swamp meadow, biodiversity, nitrogen fertilization, sustainable grassland management, trait-function relationship, winter-only grazing

## Abstract

Grazing and fertilization are major drivers of grassland ecosystem functioning, yet the mechanisms through which their interaction shapes plant functional traits and productivity remain only partially understood. We carried out a split-plot field experiment in alpine wetland meadows, combining seasonal versus year-round grazing with three nitrogen addition levels (0, 5, and 10 g N m^–2^yr^–1^). We measured community-level leaf traits, soil physicochemical properties, biodiversity metrics, and aboveground biomass (AGB), and used ordination, machine learning, and structural equation modeling. Relative to year-round grazing, winter-only grazing markedly increased community-weighted leaf area (CWM_LA; about 20% increase, P < 0.05) and generally elevated specific leaf area (SLA), shifting trait expression toward more acquisitive strategies. Fertilization responses were weaker and strongly dependent on grazing: under winter-only grazing, nitrogen inputs promoted more conservative leaf traits in grasses (reduced SLA, higher LDMC), whereas under continuous grazing, trait shifts were minimal. Principal component analysis indicated that grazing was the dominant source of trait variation, with fertilization adjusting community placement along the leaf economic spectrum. Random forest models highlighted available phosphorus, total phosphorus, soil pH, and CWM_SLA as the main positive predictors of AGB, while excessive nitrogen input showed a negative relationship with biomass. Variation partitioning revealed that management factors alone explained about 45% of biomass variation (P < 0.05), surpassing soil, traits, or diversity. Structural equation modeling showed that grazing reduced AGB indirectly by decreasing soil nutrients and plant diversity, whereas fertilization directly lowered biomass (b = –0.326, P < 0.05), likely due to acidification and altered community structure. These findings offer insight into sustainable grassland management, suggesting that adjusting grazing regimes may be more effective than fertilization in improving ecosystem functioning under non–N-limited conditions.

## 1 Introduction

Grassland ecosystems support global livestock production and hold substantial carbon stores, however, chronic overgrazing and poorly managed fertilization have decreased biodiversity and weakened ecosystem functioning (Borer et al., 2014). Owing to their isolation and fragile environments, alpine regions are particularly vulnerable to these pressures (Guan et al., 2023). Grazing—the most widespread land use in grasslands—directly alters plant community structure and productivity through defoliation and trampling, and indirectly through shifts in soil nutrient cycling and plant-microbe interactions (Li et al., 2024). Numerous studies have reported that increasing grazing intensity generally reduces plant height and the proportion of aboveground biomass while increasing the leaf nitrogen concentration and specific leaf area (SLA), which are traits linked to rapid growth and herbivory tolerance (Zhou et al., 2024). In policy terms, to protect fragile alpine ecosystems, large-scale, continuous year-round grazing is not a mainstream management practice across the core regions of the Qinghai-Tibet Plateau and is often restricted. In this study, the “AG” (annual grazing) treatment serves as a strong contrast to WG (seasonal use), aiming to reveal the fundamental influence of seasonal rest on grassland resilience. Moreover, it functions as a “stress test”, helping to rapidly identify the system’s sensitivity to external disturbances and its potential degradation thresholds. Plant responses to environmental drivers and their consequences for ecosystem functioning are strongly mediated by both resource availability and the composition of plant functional groups (Siebenkäs and Rocher, 2016; Zheng et al., 2015; Siebenkäs et al., 2017). A trait-based framework is therefore needed to clarify how different grazing regimes reorganize plant strategies and ecological processes and to inform sustainable management.

Nutrient deposition and fertilizer application reshape competitive hierarchies and diversity in grasslands (Borer et al., 2014). Eutrophication decreases species richness mainly by intensifying competition for light, a process that herbivores can partly counteract by cropping dominant species (Thomson, 2016). Cross-continental experiments have shown that nutrient enrichment typically reduces local richness, but concurrent grazing can alleviate some losses in highly productive systems (Grossman and Rice, 2014). This nutrient-herbivory interaction highlights how fertilization and grazing may exert contrasting influences on biodiversity and productivity. In practice, however, fertilization is often used to increase forage yield without matching inputs to grazing pressure; excessive nitrogen may provide little benefit when other nutrients are limiting, and can acidify soils while further reducing biodiversity (Dai et al., 2018b). High nutrient inputs often give dominance to a few competitively strong species, shifting community-level trait distributions—usually toward higher SLA and other ‘fast’ economic traits—but some taxa may instead adopt more conservative traits under nutrient excess and soil acidification. Linking grazing management with nutrient dynamics is therefore crucial for understanding how functional traits and ecological processes respond to different management strategies.

Plant functional traits—especially those organized along the Leaf Economics Spectrum (LES)—provide a unifying framework for interpreting life-history trade-offs and ecosystem processes (Wright et al., 2004).The LES spans a gradient from resource acquisitive to resource conservative strategies: acquisitive species present high SLA and leaf nitrogen concentration (LNC), thin and short-lived leaves, and rapid growth (Bektaş et al., 2025); conservative species present thick, dense leaves with high leaf dry matter content (LDMC) and low SLA, long leaf lifespans, and slow resource turnover, improving stress tolerance (Halbritter et al., 2025). Globally, the leaf traits of vascular plants tend to follow this coordinated variation. Grazing and nutrient addition are expected to shift communities along the LES axis: grazing pressure often favors short, grazing-tolerant species leaning toward conservative strategies, whereas nutrient enrichment supports fast-growing species (Henn et al., 2024). Yet the specific regime matters. Restricting grazing to the non-growing season (winter-only grazing) may protect vegetation from continuous defoliation during peak growth. This practice can enable graminoids to increase leaf area and accumulate biomass, a response consistent with strategic grazing management that promotes grassland multifunctionality (Wang et al., 2019). Such shifts in biomass allocation and leaf morphology may reflect broader plant economic strategies, aligning with trait variations described along the worldwide Leaf Economics Spectrum (Wright et al., 2004) and within the integrated spectrum of plant form and function (Díaz et al., 2016). Concurrently, by removing litter and senesced material, winter-only grazing may influence nutrient mineralization rates and subsequent spring productivity. These proposed mechanisms—particularly their combined effects and context-dependent outcomes in alpine wetland meadows—still require further empirical evaluation. However, critical knowledge gaps remain regarding how seasonal grazing regimes (annual vs. winter grazing) modulate community-level responses to nutrient enrichment through shifts in plant leaf economic spectrum strategies. Furthermore, the specific pathways and relative contributions by which grazing and fertilization interactively regulate productivity—via modifications to soil properties (e.g., available phosphorus, pH) and plant functional traits—remain poorly understood. Addressing these gaps is essential for developing sustainable grassland management strategies in the context of increasing nitrogen deposition.

Against this background, we focus on two central levers of grassland management—grazing regime and nitrogen addition. We established a split-plot experiment in an alpine swamp meadow on the Qinghai–Tibetan Plateau, combining year-round and winter-only grazing with three levels of nitrogen addition, to evaluate both main and interactive effects. By measuring key leaf functional traits and applying multivariate analyses, we test the hypotheses that winter-only grazing, by avoiding defoliation in the growing season, shifts communities toward more acquisitive leaf traits and greater trait diversity, promotes soil nutrient buildup, and thereby indirectly enhances productivity; in contrast, year-round grazing weakens these pathways. We also expect that moderate fertilization will raise biomass where nitrogen is limiting, whereas when nitrogen is not the principal constraint—or becomes excessive—soil acidification and compositional change will decrease any positive effects on productivity. Our aim is to clarify, through functional traits and soil processes, the mechanisms through which grassland production responds to management. We hypothesized that, given the potential phosphorus limitation in this alpine meadow, nitrogen addition alone would have limited or even negative effects on plant productivity, potentially mediated by soil acidification or nutrient imbalance. However, we expected that winter grazing could mitigate these negative effects by improving soil conditions (e.g., alleviating acidification, increasing phosphorus availability) and promoting acquisitive plant functional traits, thereby enhancing community productivity. By contrast, annual grazing was predicted to constrain such positive responses due to chronic disturbance and limited seasonal recovery. Our aim was to elucidate, through integrated measurements of plant functional traits and soil processes, the mechanisms by which grazing seasonality and nitrogen addition interactively regulate grassland productivity. To achieve this, we address the following three questions: (1) How does seasonal (winter-only) grazing alter functional trait composition relative to year-round grazing? (2) Does the influence of nitrogen addition on plant traits and community structure depend on grazing regime? (3) Through which pathways do grazing and fertilization affect aboveground productivity? The study is designed to provide an evidence base for strategies that jointly maintain productivity and biodiversity.

## 2 Materials and methods

### 2.1 Study site

**Figure 1:**
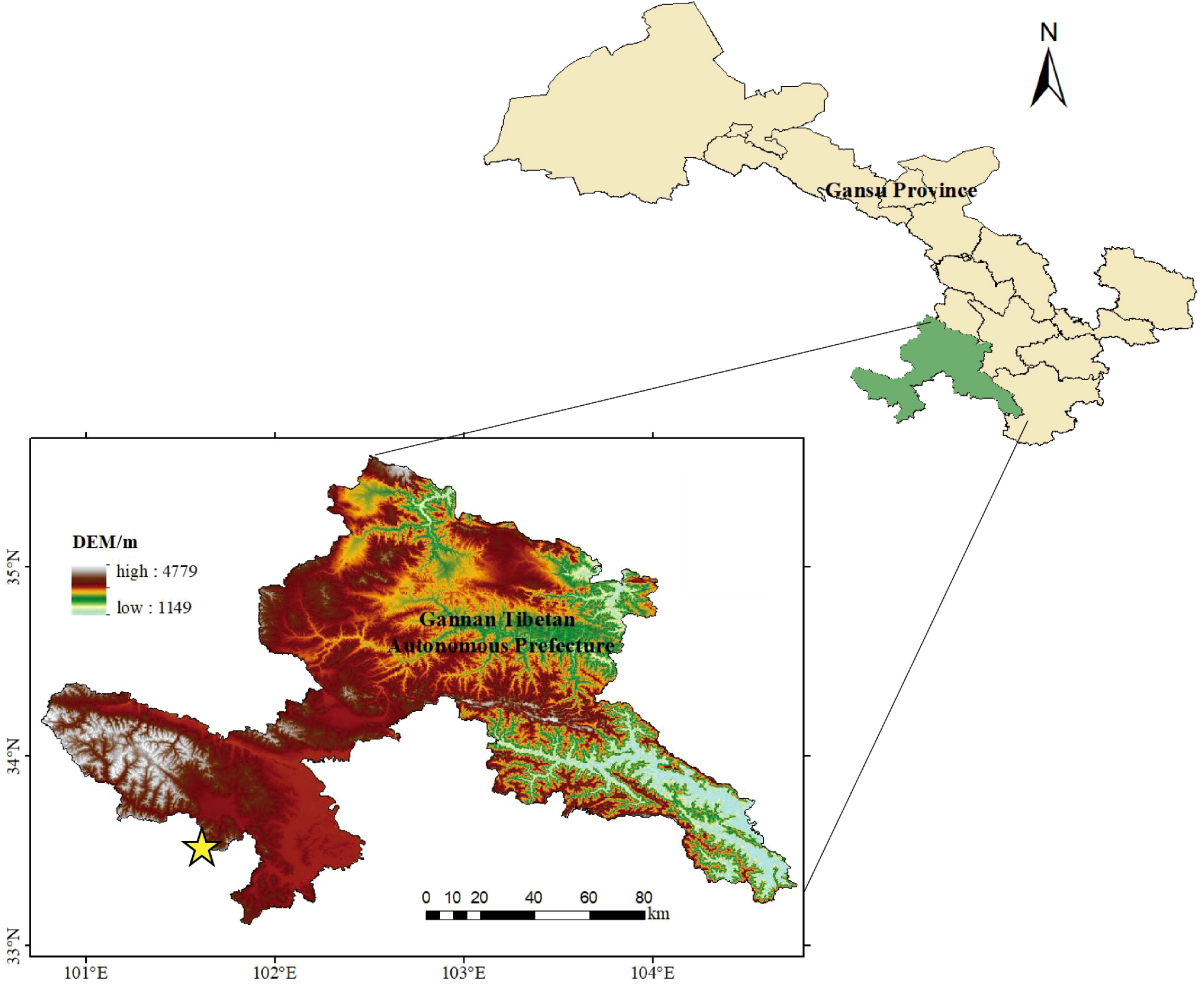
Overview of the study area.

The experiment was conducted in Maqu County, Gannan Tibetan Autonomous Prefecture, Gansu Province, China, at the Alpine Meadow and Wetland Ecosystem Research Station of Lanzhou University (33°39′N, 101°53′E; elevation 3,650 m) (**Figure 1**). The region has a cold semihumid to semiarid climate, with a mean annual temperature of 2.2 °C and an average annual precipitation of about 620 mm, most of which falls between July and August. The vegetation is dominated by subalpine meadow communities composed of Poaceae, Cyperaceae, and various Forbs. The study plots were located along the marginal zone of a slightly degraded marsh meadow, characterized by seasonally shallow water retention. This hydrological pattern and floristic composition differ systematically from those of intact marsh meadows, providing a stable ecological background for examining how grazing and nitrogen enrichment regulate ecosystem functioning. According to previous studies in the same region, the background soil total nitrogen content in alpine meadows ranges from approximately 4.5 to 6.5 g kg^−1^, with soil available nitrogen (NH_4_^+^-N + NO_3_^−^-N) averaging 49.4 mg kg^−1^ in lightly degraded grasslands. The estimated regional background atmospheric nitrogen deposition rate is approximately 8–15 kg N ha^−1^ yr^−1^ (0.8-1.5 g N m^−2^ yr^−1^) (Yan et al., 2010).

### 2.2 Experimental design

We used a split-plot randomized block design to separate the main and interactive effects of grazing regime and nitrogen enrichment. The whole-plot factor consisted of two grazing treatments: year-round grazing (AG) was characterized by moderate to high utilization rates (50–70%), a stocking rate of approximately 3.5–3.9 head hm^−2^ yr^−1^, and a grazing area of 0.61 hm^2^. In contrast, winter-only grazing (WG) involved restricting livestock access during the growing season (May to September), with grazing permitted only from October to April each year. This treatment had a stocking rate of 3.4–3.8 head hm^−2^ yr^−1^ and a grazing area of 0.51 hm^2^. Within each grazing regime, subplots were randomly assigned one of three nitrogen addition levels: control (CK, 0 g N m^–2^yr^–1^), low nitrogen (LN, 5 g N m^–2^ yr^–1^), and high nitrogen (HN, 10 g N m^–2^ yr^–1^), with six replicates per treatment, resulting in a total of 36 plots (2 grazing × 3 fertilization × 6 replicates; plot size: 5 m × 5 m). Nitrogen fertilizer was applied as a single annual application in early May at the beginning of the growing season. The data presented in this study are based on observations following two consecutive years (2022–2023) of nitrogen addition treatments.

Nitrogen was applied as urea [CO(NH_2_)_2_] via surface broadcasting during overcast or rainy conditions in mid to late May to reduce volatilization and improve solubility and infiltration. To maintain spatial independence and minimize autocorrelation, the two experimental blocks were placed 20 m apart along the outer wetland boundary.

### 2.3 Plant survey and aboveground biomass

During peak biomass accumulation in August, a 50 cm × 50 cm quadrat was randomly positioned within each plot to assess the species composition, total and relative cover, and mean canopy height. Species richness was recorded as the number of taxa present within the quadrat. After vegetation surveys, all aboveground biomass within the quadrat was clipped at ground level, weighed fresh, and then oven-dried at 60 °C to constant mass. The dry biomass was standardized by area and expressed as aboveground biomass (AGB; g m^–2^).

### 2.4 Plant functional traits

Between late July and mid-August, for each target species within a plot, we collected ≥5 mature, healthy, and fully expanded sun-exposed leaves from different individuals. Individuals were randomly selected from a representative area of the plot (avoiding edges and microsites) to capture average community-level trait variation. All sampled individuals were at similar phenological stages (peak growing season). Leaves of the same species and plot were pooled for specific leaf area (SLA) and leaf dry matter content (LDMC) measurements following standardized protocols (Pérez-Harguindeguy et al., 2013). Three core traits of the leaf economic species were measured: the leaf area (LA), which was determined by scanning leaf images and estimating area using ImageJ; specific leaf area (SLA, m^2^ g^–1^), which was calculated as the leaf area divided by the dry mass; and the leaf dry matter content (LDMC, g g^–1^), which was calculated as the dry mass divided by the fresh mass. At the community level, trait values were aggregated using community-weighted means (CWM), which were computed by weighting species-level traits by their relative cover or biomass in each plot. The CWM for a given trait was calculated as follows:


$$CWM=\sum pi\times trait_{i}$$

where p_i_ denotes the relative productivity of species i, and trait_i_ is the trait value of species i within the plot.

### 2.5 Soil sampling and physicochemical analysis

After the vegetation surveys, three soil cores (0–20 cm depth) were collected from the center and corners of each quadrat and combined into a single sample. Soils were sampled from the 0–20 cm depth using a stainless steel auger (5 cm diameter). This depth was chosen because previous studies in alpine meadows on the Qinghai-Tibet Plateau have shown that approximately 70-90% of root biomass is concentrated in the top 20 cm (Yang et al., 2009), representing the primary zone of nutrient uptake and plant-soil interactions. The 0–20 cm composite layer is also widely used in regional studies, facilitating direct comparison with existing literature. Samples were air-dried, cleared of debris, and sieved through a 2 mm mesh before analysis. Soil pH was measured in a 1:2.5 soil-to-water suspension using a glass electrode, and electrical conductivity (EC) was determined in a 1:5 soil-to-water extract. Gravimetric soil moisture content was assessed by oven-drying at 105 °C.

Total nitrogen (TN) was measured via Kjeldahl digestion, and total phosphorus (TP) was analyzed following H_2_SO_4_–HClO_4_ digestion using molybdenum-antimony colorimetry. Soil organic carbon (SOC) was determined by external heating oxidation with potassium dichromate. Available phosphorus (AP) was extracted with NaHCO_3_ and quantified colorimetrically, while available potassium (AK) was extracted using 1 mol L–1 NH_4_OAc and measured by flame photometry. Ammonium (NH_4_^+^–N) and nitrate (NO_3_–N) concentrations were determined using a flow injection analyzer.

### 2.6 Soil microbial sequencing

Total genomic DNA was extracted using MagPure Soil DNA LQ Kit (Magan) following the manufacturer’s instructions. DNA concentration and integrity were measured with NanoDrop 2000 (Thermo Fisher Scientific, USA) and agarose gel electrophoresis. Extracted DNA was stored at-20 °C until further processing. The extracted DNA was used as template for PCR amplification of bacterial 16S rRNA genes with the barcoded primers and Takara Ex Taq (Takara). For bacterial diversity analysis, V3-V4 (or V4-V5) variable regions of 16S rRNA genes was amplified with universal primers 343F (5’-TACGGRAGGCAGCAG-3’) and 798R (5’-AGGGTATCTAATCCT-3’)1 [or 515F (5’-GTGCCAGCMGCCGCGG-3’) and 907R (5’-CCGTCAATTCMTTTRAGTTT-3’) 2 for V3-V4 regions [or V4-V5 regions].The Amplicon quality was visualized using agarose gel electrophoresis. The PCR products purified with AMPure XP beads (Agencourt) and amplified for another round of PCR. After purified with the AMPure XP beads again, the final amplicon was quantified using Qubit dsDNA Assay Kit (Thermo Fisher Scientific,USA). The concentrations were then adjusted for sequencing. Sequencing was performed on an Illumina NovaSeq 6000 with 250 bp paired-end reads. (Illumina Inc., San Diego, CA; OE Biotech Company; Shanghai, China).

### 2.7 Statistical analysis

All statistical analyses were conducted in R (version 4.2.2). Prior to analysis, data were checked for normality using the Shapiro-Wilk test and for homogeneity of variances using Levene’s test. No severe violations of these assumptions were detected (all P > 0.05), justifying the use of parametric tests. For principal component analysis (PCA), all trait variables were Z-score standardized to account for differences in measurement scales, ensuring that traits with larger units did not disproportionately influence the ordination results. To assess the main and interactive effects of management treatments on response variables, we performed a Type-III analysis of variance using the car package, followed by *post hoc* pairwise comparisons using the emmeans package. Principal component analysis of community-weighted trait means was performed with FactoMineR, and treatment effects on multivariate trait space were evaluated using Type-III ANOVA and PERMANOVA (via the vegan package), with effect sizes reported as partial η^2^.

To quantify the predictive importance and explanatory power of ecological factors on aboveground biomass, a random forest regression model was built with the ranger package (1000 trees). Variable importance was assessed via permutation, and bootstrap resampling was used to estimate uncertainty. Predictors were grouped into functional categories (traits, soil, biodiversity, management, etc.), and group-level contributions were calculated. Partial dependence plots were generated to visualize nonlinear responses and thresholds. Variation partitioning analysis (using vegan) was then applied to evaluate the unique and shared explanatory power of each group, with significance tested via permutation and confidence intervals derived from bootstrap distributions.

To resolve the pathways through which management influences aboveground biomass, we constructed a structural equation model based on *a priori* ecological hypotheses, implemented in lavaan. The model included direct effects from soil properties and plant traits and allowed for indirect management effects. Model estimation was performed using robust maximum likelihood, and standardized path coefficients with bootstrap confidence intervals were used to evaluate significance. Model selection was based on fit indices and ecological interpretability, and the final path diagram was visualized using semPlot. All figures were generated using ggplot2 package.

## 3 Results

Compared to year-round grazing (AG), winter-only grazing (WG) significantly enhanced leaf area (LA) in all three functional groups, including Poaceae (F = 77.82, P< 0.05), Cyperaceae (F = 18.10, P< 0.05), and forbs (F = 10.08, P< 0.05). This consistent positive effect across functional groups indicates that grazing regime was the primary driver of LA variation, with WG promoting greater leaf expansion regardless of plant taxonomy.

In contrast, fertilization produced comparatively weak main effects. Significant responses to nitrogen addition were detected only for leaf dry matter content (LDMC) in Cyperaceae (F = 6.26, P = 0.005), and this effect was primarily observed under WG rather than AG. Specific leaf area (SLA) responses to grazing were taxon-specific: WG significantly increased SLA in forbs and Cyperaceae (F = 66.86 and 8.17, respectively; both P< 0.05) but had no significant effect on SLA in Poaceae (**Supplementary Figure 1**). This suggests that the magnitude of trait plasticity in response to grazing seasonality varies among functional groups, with Poaceae exhibiting greater stability in SLA.

At the community level, WG markedly increased community-weighted mean leaf area (CWM_LA) across all three functional groups (P< 0.001). This result reinforces the role of winter grazing in enhancing overall community leaf display. By contrast, CWM_SLA and CWM_LDMC showed fertilization responses that were strongly contingent upon grazing regime, revealing a clear interactive effect between the two treatments.

**Figure 2:**
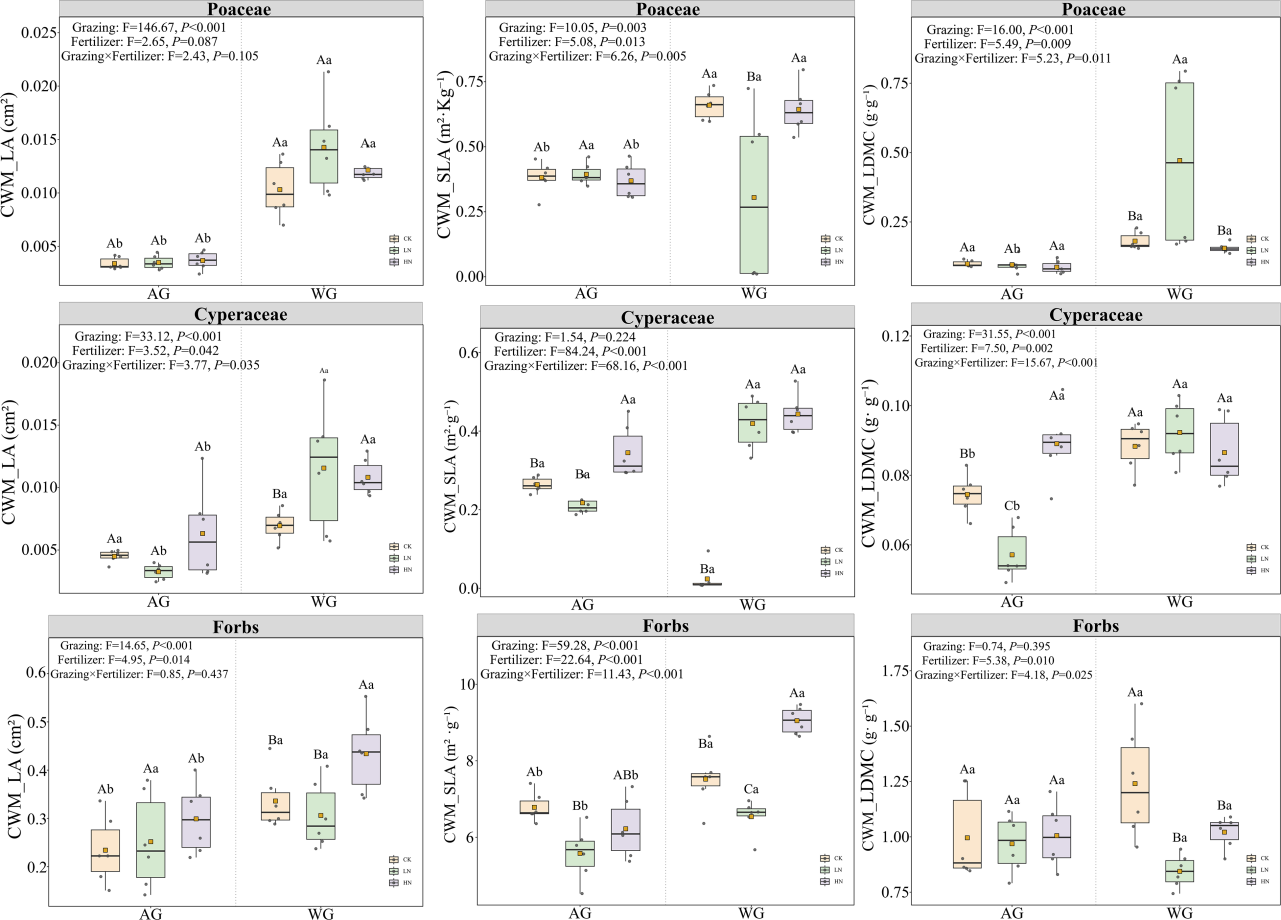
Trait-level responses of leaf economic spectrum to grazing and fertilization. The figure shows community-weighted leaf area (CWM_LA), specific leaf area (CWM_SLA), and leaf dry matter content (CWM_LDMC) across three functional groups (Poaceae, Cyperaceae, and Forbs) under different grazing regimes (AG, WG) and fertilization levels (CK, LN, HN). Different uppercase letters indicate significant differences among fertilization treatments (Tukey HSD, *P* < 0.05), while different lowercase letters indicate significant differences between grazing regimes (Tukey HSD, *P* < 0.05).

In Poaceae, both CWM_SLA and CWM_LDMC exhibited significant grazing × fertilization interactions (P< 0.01). Specifically, the effect of fertilization was only evident under WG, where low nitrogen (LN) addition reduced CWM_SLA and increased CWM_LDMC relative to control. This pattern indicates that under winter grazing, low-level nitrogen addition promotes a shift toward more conservative resource-use strategies in grasses. In Cyperaceae, all three community-weighted traits displayed significant interactive effects (P< 0.05). Notably, CWM_SLA reached its highest value under the combination of annual grazing and high nitrogen (AG+HN), while remaining elevated under both WG+LN and WG+HN compared to control. For CWM_LDMC, the response to fertilization followed a distinct pattern under AG (HN > CK > LN), whereas under WG, no significant fertilization effects were detected. For forbs, both CWM_SLA and CWM_LDMC were jointly shaped by grazing regime, nitrogen addition, and their interaction. Under AG, CWM_SLA followed the order CK > HN > LN, whereas under WG the pattern reversed to HN > LN > CK (P< 0.05). Correspondingly, the combination of WG and no fertilization (WG+CK) produced the highest CWM_LDMC, surpassing both LN and HN treatments under the same grazing regime. These contrasting patterns suggest that fertilization under different grazing regimes fundamentally reconfigures community-level functional strategies in forbs (**Figure 2**).

### 3.1 Trait spectrum shifts along orthogonal axes: grazing dominates PC1, fertilization modulates acquisitive-conservative positioning.

**Figure 3:**
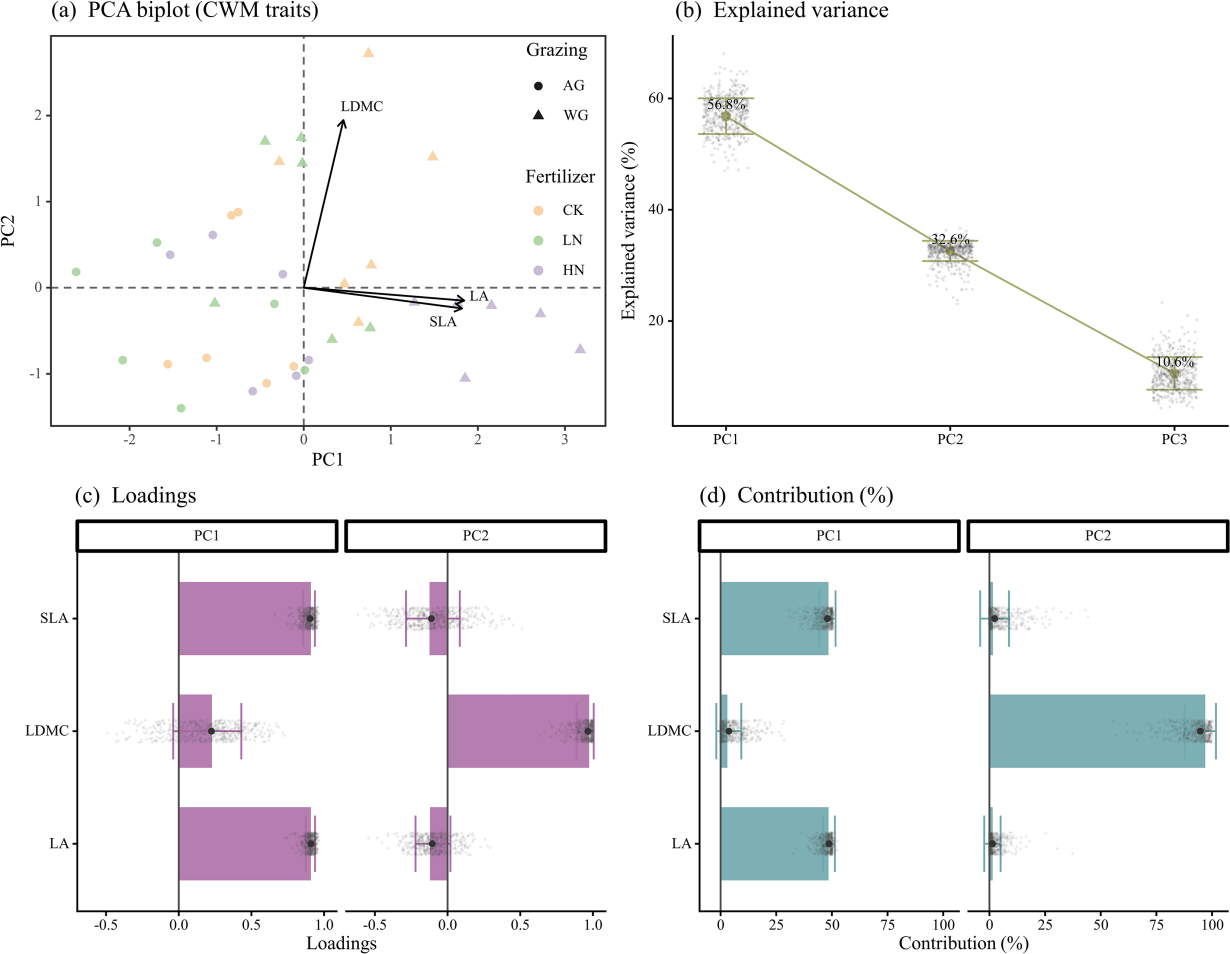
PCA of community-weighted leaf traits and uncertainty assessment. **(a)** Biplot of principal component analysis (PCA) based on three standardized community-weighted leaf traits: leaf area (LA), specific leaf area (SLA), and leaf dry matter content (LDMC). Points represent individual plots (color-coded by fertilization level: CK, LN, HN; shape-coded by grazing regime: AG, WG), and arrows denote trait loading vectors. **(b)** Scree plot showing the proportion of variance explained by each principal component. Thick lines represent observed values; error bars reflect bootstrap standard deviations (n = 499); grey dots show bootstrap distributions. Percentages indicate variance explained by each axis. **(c)** Trait loadings on PC1 and PC2. Bars indicate observed loadings; dots and error bars represent bootstrap estimates and uncertainty. **(d)** Trait contributions to PC1 and PC2. Values indicate the relative role of each trait in defining the component axes.

Principal component analysis (PCA) integrated the three community-weighted leaf traits (LA, SLA, LDMC) into two orthogonal axes (**Figure 3a**), cumulatively explaining 89.4% of total variance (PC1 = 56.8%, PC2 = 32.6%; **Figure 3b**). Trait loadings showed that PC1 was driven mainly by SLA, with LA contributing secondarily, while PC2 captured variation in LDMC (**Figures 3c, d**), representing a typical gradient along the leaf economic spectrum.

Treatment effects were strongest along PC1. Type-III ANOVA identified highly significant main effects of grazing (F = 62.67, *P* < 0.001, partial η^2^ = 0.68) and fertilization (F = 14.44, *P* < 0.001, partial η^2^ = 0.49), with a moderate interaction effect (F = 4.00, *P* = 0.029, partial η^2^ = 0.21; **Supplementary Table 1**). By contrast, PC2 responded moderately to grazing (F = 6.03, *P* = 0.020, partial η^2^ = 0.17), but showed no significant fertilization or interaction effects (*P* > 0.10; **Supplementary Table 1**). At the multivariate scale, PERMANOVA confirmed significant treatment-driven divergence in trait space (overall model: F = 9.11, R^2^ = 0.60, *P* = 0.001), consistent with the dominant effect observed along PC1. Taken together, grazing exerted a stronger influence than fertilization on community placement along the leaf economic spectrum. Fertilization mainly adjusted community strategy via displacement along PC1, shifting expression between acquisitive and conservative syndromes.

### 3.2 Relative contributions of community function, soil environment, and management to biomass

**Figure 4:**
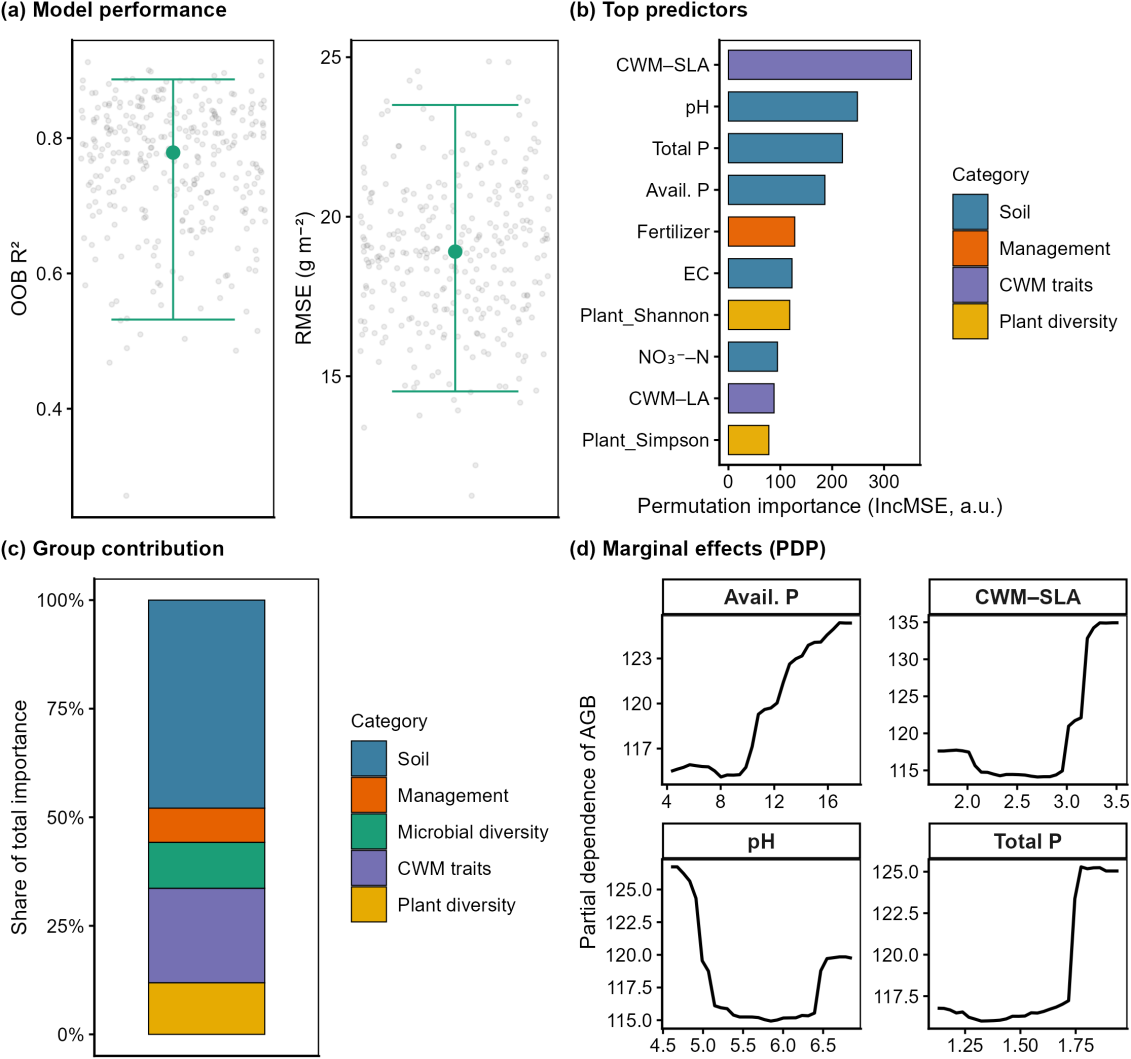
Performance and variable contributions of the random forest model predicting aboveground biomass. **(a)** Model performance metrics: distribution of out-of-bag R^2^ and root mean square error (RMSE) across bootstrap iterations. Small dots represent individual replicates; large dots and error bars indicate median values and 95% quantile ranges. **(b)** Top 10 predictors ranked by permutation-based importance. Values represent the mean decrease in model accuracy when each variable is randomly permuted. **(c)** Relative contributions of grouped ecological variables to AGB prediction. Categories include soil properties, plant functional traits, biodiversity metrics, management factors, and microbial diversity. **(d)** Partial dependence plots (PDPs) for the four most influential predictors, showing marginal effects on AGB across observed gradients. Curves represent smoothed responses; shaded regions denote bootstrap uncertainty.

Random forest (RF) modeling produced reliable and precise estimates of aboveground biomass (AGB), with an out-of-bag R^2^ of 0.77 and a root mean square error (RMSE) of 18.9 g m^–2^ (**Figure 4a**). Among all predictors, community-weighted specific leaf area (CWM_SLA) was identified as the most influential variable (mean permutation importance = 352.8), followed by soil characteristics including pH, total phosphorus (TP), and available phosphorus (AP). Management factors, such as fertilization and plant diversity indicators, such as the Shannon index, displayed moderate contributions (**Figure 4b**). Analysis of grouped variables further clarified the relative roles of ecological components: soil attributes represented the largest portion of predictive strength (47.9%), with plant functional traits (21.8%) and plant diversity (11.9%) contributing less. Microbial diversity and management factors showed comparatively minor effects (**Figure 4c**). Partial dependence plots (PDPs) highlighted nonlinear biomass responses to soil nutrient gradients: AGB increased with AP and TP but reached a plateau at higher concentrations, while pH exhibited a bidirectional pattern (**Figure 4d**).

To separate the independent contributions of each ecological group, we performed variation partitioning analysis (VPA; **Supplementary Figure 2**). Overall, the four variable groups explained 63.5% of the variance in AGB (adjusted R^2^ = 0.635). Management factors exhibited the highest unique explanatory power (adjusted R^2^ = 0.454, *P* = 0.003), remaining significant after controlling for other groups. By contrast, the individual contributions of CWM traits, soil properties, and biodiversity were not statistically significant (*P* > 0.05). Shared variance across groups accounted for 14.7%, and residual unexplained variation remained at 36.5% (**Supplementary Figure 2**).

### 3.3 Grazing acts indirectly via soil-diversity pathways, whereas fertilization directly reduces AGB

**Figure 5:**
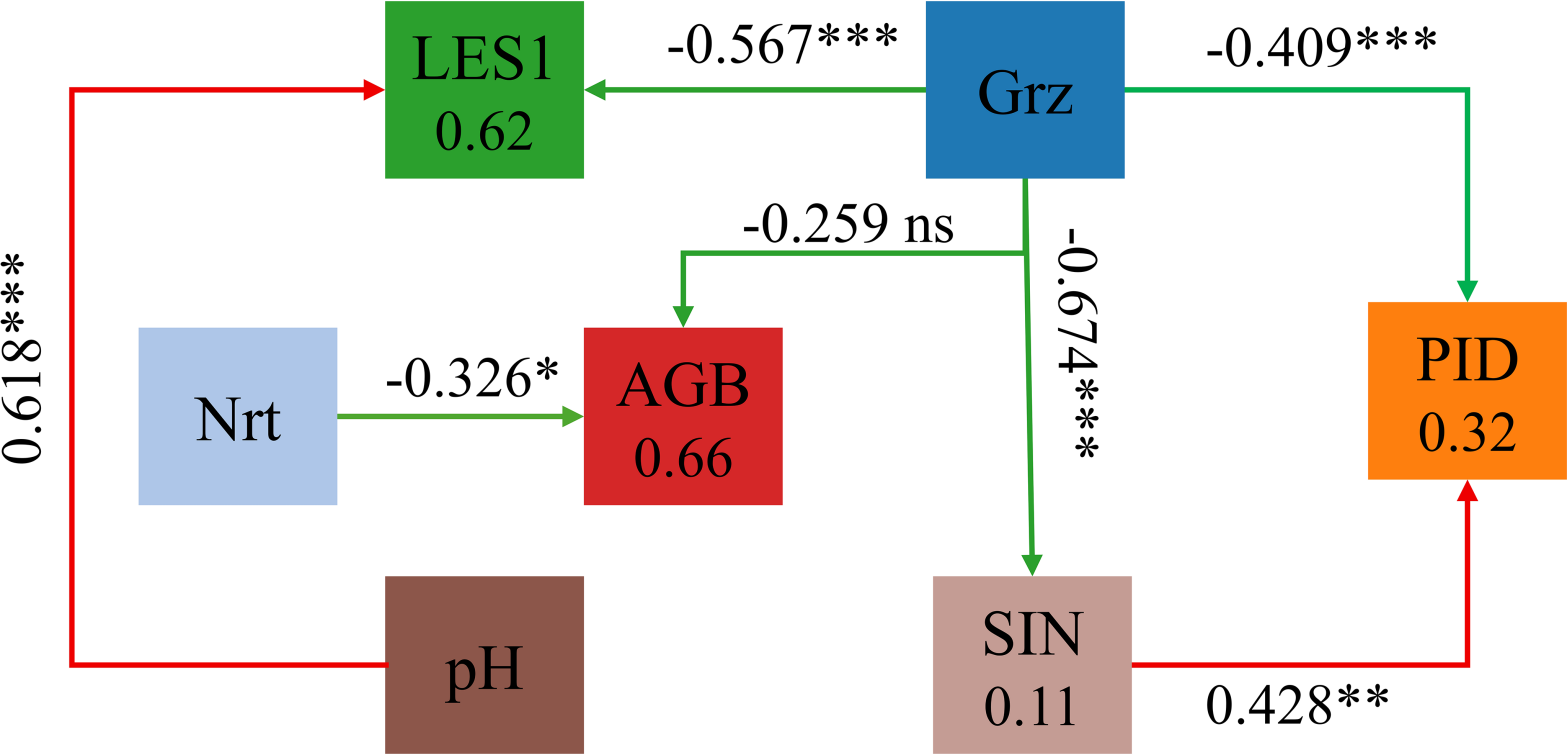
Relative influence of grazing and fertilization on aboveground biomass. Arrows represent standardized path coefficients, with red indicating positive effects and green indicating negative effects. Asterisks denote significance levels (*P < 0.05; **P < 0.01; ***P < 0.001; ns = not significant). Squares show variable names and R^2^ values. Variables include grazing intensity (Grz), nitrogen fertilization rate (Nrt), soil pH (pH), soil nutrient index (SIN), leaf economic spectrum (LES), plant species diversity (PID), and aboveground biomass (AGB). SIN represents a composite measure of key soil characteristics, including pH, total phosphorus (TP), and available phosphorus (AP). PID incorporates both the Shannon and Simpson diversity indices derived from plant community data. SEM fit indices: χ^2^ = 3.87, df = 4, *P* = 0.42; RMSEA = 0.021; CFI = 0.998; SRMR = 0.024.

Structural equation modeling (SEM) showed that grazing intensity (Grz) had significant negative effects on the leaf economic spectrum axis (LES), plant community diversity (PID), and soil nutrient index (SIN) (standardized path coefficients: β = –0.567, –0.409, and –0.674 respectively; *P* < 0.05), while its direct effect on aboveground biomass (AGB) was not significant (**Figure 5**). SIN positively influenced PID (β = 0.428; *P* < 0.05), forming an indirect pathway from Grz to PID through SIN. The indirect effect was –0.288, and the total effect of Grz on PID reached –0.697, indicating that grazing suppresses ecosystem functioning through linked soil-community structural pathways.

Fertilization intensity (Nrt) also had a significant direct negative effect on AGB (β = –0.326; *P* < 0.05), independent of soil or diversity mediation. Additionally, soil pH significantly enhanced LES expression (β = 0.618; *P* < 0.05), suggesting that edaphic conditions regulate trait syndromes at the community scale.

## 4 Discussion

### 4.1 Reshaping of the leaf economics spectrum and productivity pathways by grazing seasonality in alpine wetland meadows

In this study, we identify the grazing regime as the primary driver of community dynamics and ecosystem functioning, with effects that clearly outweigh those of nitrogen addition. Relative to year-round continuous grazing (AG), seasonal winter-only grazing (WG) significantly increased community-weighted leaf area (CWM_LA; ≈20%, *P* < 0.05) and generally elevated specific leaf area (SLA), thereby shifting the community leaf economics spectrum (LES) toward more resource-acquisitive strategies. This aligns with ecological theory, suggesting that intermediate disturbance can optimize resource allocation and encourage species coexistence, ultimately supporting functional diversity and ecosystem productivity (Nguyen et al., 2014; Yue et al., 2025; Yang et al., 2023). By offering a disturbance-free recovery period during the growing season, winter-only grazing allowed grasses, sedges, and forbs to expand leaf area and accumulate biomass, developing larger leaves with higher SLA. Such trait adjustments reflect improved photosynthetic capacity and resource acquisition at the community level and, in turn, contribute to higher primary productivity (Halbritter et al., 2025).

In contrast, year-round grazing, due to persistent defoliation pressure, shifts the community LES toward conservative strategies, evident in higher leaf dry matter content (LDMC) and reduced leaf area. This result accords with classic grazing theory, where intense grazing filters species with thicker, denser tissues and slower growth that are better able to deter herbivory (Zhou et al., 2024; Vuorinen et al., 2021). Functional groups, however, differed in their responses: under winter-only grazing, SLA rose sharply in Cyperaceae and forbs, whereas Poaceae remained comparatively stable. This may relate to the strong regenerative capacity and tolerance mechanisms associated with grasses (Zheng et al., 2015), while sedges and forbs take advantage of the disturbance-free growing season to enhance competitive performance (Li et al., 2018).

Accordingly, winter-only grazing creates an “ecological window,” assembling a trait configuration that favors growth and ultimately increases community-level productivity—offering a nuanced perspective on how alpine grasslands maintain resilience under disturbance (Wu et al., 2016; Li et al., 2019). These findings advance understanding of alpine wetland responses to grazing pressure and provide empirical grounding for theoretical frameworks aiming to sustain grassland ecosystem functioning under global change.

### 4.2 Context dependence and potentially negative direct effects of nitrogen addition

Our study shows that the effects of nitrogen addition on grassland ecosystems are strongly context dependent and can become negative under particular conditions. Under the year-round grazing (AG) scenario, medium to high nitrogen inputs did not significantly increase aboveground biomass (AGB) or shift community functional traits. This suggests that, under continuous grazing pressure, added nutrients may fail to generate productivity gains. Continuous grazing can reduce vegetation cover and height, allowing nutrients to be lost or poorly absorbed, thereby lowering nutrient use efficiency (Xiang et al., 2023). The random forest analysis provides critical insight into the underlying nutrient limitation regime at our study site: available phosphorus (AP) and total phosphorus (TP) emerged as the dominant positive predictors of AGB, whereas nitrogen addition showed comparatively low importance and was negatively associated with AGB. This finding strongly suggests that primary productivity in this alpine meadow is primarily limited by phosphorus rather than nitrogen—a pattern increasingly recognized in alpine ecosystems on the Qinghai-Tibet Plateau, where low temperatures and slow mineralization rates constrain phosphorus availability (Bing et al., 2016). Under such P-limited conditions, the addition of nitrogen alone is unlikely to stimulate productivity and may even exacerbate nutrient imbalances by increasing the N:P ratio beyond optimal ranges for plant growth (Peñuelas et al., 2013). The lack of positive biomass response to nitrogen addition under AG, coupled with the negative association between nitrogen and AGB in the random forest model, can therefore be explained by the existing P limitation: without concurrent phosphorus supply, added nitrogen fails to alleviate the primary growth-limiting factor and may instead induce secondary stress. This interpretation is consistent with global-scale evidence that single-nutrient nitrogen additions rarely produce sustained biomass increases in P-limited ecosystems (Fay et al., 2015; Harpole et al., 2016). F = 8.67, P< 0.001.

Under the winter-only grazing (WG) treatment, nitrogen addition reduced SLA and increased LDMC in Poaceae communities, indicating that fertilization promoted conservative trait expression. This may occur because fertilization favors species that tolerate nutrient enrichment and herbivory (e.g., perennial rhizomatous grasses or taxa with lower nutrient requirements), shifting community mean traits toward the conservative end of the leaf economic spectrum (LES). Notably, this trait shift toward conservatism under nitrogen addition—despite the presence of P limitation—suggests that even when nitrogen is not the primary limiting nutrient, it can still act as a selective pressure reshaping community functional composition. However, this compositional shift did not translate into productivity gains, reinforcing the conclusion that nitrogen alone is insufficient to enhance biomass in this P-limited system.

Structural equation modeling further showed a significant direct negative effect of nitrogen addition on AGB (β = –0.326), suggesting that excess nitrogen directly suppresses yield. In P-limited ecosystems, several mechanisms could account for this negative effect. First, nitrogen addition may exacerbate N:P stoichiometric imbalance, leading to phosphorus depletion and reduced growth efficiency (Vitousek et al., 2010). Second, increased nitrogen availability can shift plant community composition toward species with higher nitrogen demand but lower phosphorus use efficiency, ultimately constraining biomass production (Craine et al., 2002). Third, while soil acidification has been proposed as a mechanism for nitrogen-induced yield reductions in some systems (Dai et al., 2018a; Leff et al., 2015; Ghavi-Helm et al., 2014; Xu et al., 2025), we did not present direct evidence of significant pH decline in our results; therefore, acidification remains a plausible but unverified mechanism that warrants further investigation with longer-term soil monitoring.

Taken together, our results indicate that isolated nitrogen inputs are not a consistent yield-enhancing strategy in this alpine meadow and may be detrimental when nitrogen is not the primary limiting factor. The strong predictive power of soil phosphorus variables, combined with the weak or negative effects of nitrogen addition, points to phosphorus limitation as the key constraint on productivity. These findings highlight the importance of assessing baseline nutrient limitation status before implementing fertilization programs and suggest that sustainable management in this region should consider phosphorus availability alongside nitrogen inputs. Future studies should directly manipulate both nitrogen and phosphorus to confirm their interactive effects and elucidate the mechanisms underlying negative nitrogen responses.

### 4.3 Integrated regulation of ecosystem functioning by management practices

Our findings indicate that soil physicochemical properties, particularly available phosphorus (AP) and total phosphorus (TP), play a central role in explaining aboveground biomass, thereby supporting the hypothesis of multiple nutrient co-limitation (Du et al., 2020). Structural equation modeling further showed that year-round grazing indirectly reduced productivity by lowering soil nutrient availability (β = –0.674) and community diversity (β = –0.409), producing a total negative effect of roughly –0.70. This aligns with the conclusion that overgrazing depletes soil nutrient pools and simplifies community structure (Li et al., 2023; Ren et al., 2024). In contrast, winter-only grazing, by offering a recovery period in the growing season, supported litter accumulation and decomposition, helping maintain higher soil fertility and species diversity and preventing cascading declines in ecosystem functioning. Notably, nitrogen addition in this study did not improve productivity and instead showed a direct negative influence, indicating that in alpine swamp meadow where nitrogen is not limiting, excessive N inputs may lead to soil acidification and undesirable shifts in community structure. Taken together, the results demonstrate that adjusting grazing practices (such as seasonal or rotational grazing) is more effective than fertilization alone for increasing grassland productivity, maintaining biodiversity, and supporting soil quality, thereby offering important guidance for sustainable grassland management.

It is acknowledged that ecosystem processes, particularly changes in soil properties and long-term community dynamics, operate on timescales exceeding two years. Nonetheless, this study captures the initial and significant directional responses of plant functional traits and productivity to the experimental treatments, providing a critical foundation for understanding the underlying mechanisms and informing future long-term investigations. However, the short duration of the experiment (two years) and the limited focus on microbial processes place constraints on the broader interpretation of the findings. Future studies should include long-term monitoring of ecological changes, with particular emphasis on soil microbial communities and plant–soil interactions and assess the potential of multi-element management approaches to strengthen grassland resilience under ongoing environmental change. Combining extended observations with mechanistic understanding will be essential for developing management strategies that promote more stable and sustainable alpine swamp meadow ecosystems.

## 5 Conclusion

Our results show that the timing of grazing disturbance—rather than nutrient enrichment—plays the dominant role in shaping plant functional strategies and ecosystem productivity in alpine wetland meadows. Our integrated analysis suggests that winter grazing may enhance alpine meadow productivity primarily by fostering a community-level shift towards acquisitive leaf traits (e.g., higher SLA) and by improving soil phosphorus availability. In contrast, annual grazing appeared to constrain these positive pathways. These changes collectively enhance aboveground biomass through indirect soil-diversity-function pathways. In contrast, year-round grazing imposes persistent defoliation that drives communities toward conservative trait syndromes, nutrient loss, and reduced productivity. Moreover, nitrogen addition produced a direct negative effect on biomass, likely tied to soil acidification and phosphorus limitation, highlighting that excessive N inputs can be counterproductive in non-N-limited systems. Overall, our findings show that optimizing grazing seasonality—not simply applying more fertilizer—is the most effective strategy to balance productivity and ecological integrity in alpine grasslands. This mechanistic understanding offers a scalable foundation for sustainable grassland management under accelerating global change.

## Data Availability

The raw data supporting the conclusions of this article will be made available by the authors, without undue reservation.
