# Supplementary material for: Winter-only grazing governs plant leaf economic traits and productivity pathways, with fertilization effects contingent on grazing regime
Source: Front Plant Sci. 2026 Mar 25;17:1777082. doi: 10.3389/fpls.2026.1777082 (PMC13058950; doi:10.3389/fpls.2026.1777082)
Supplement: Supplementary file 1 [file Supplementaryfile1.docx]

Supplementary Material

# Supplementary Figures and Tables

## Supplementary Figures

**
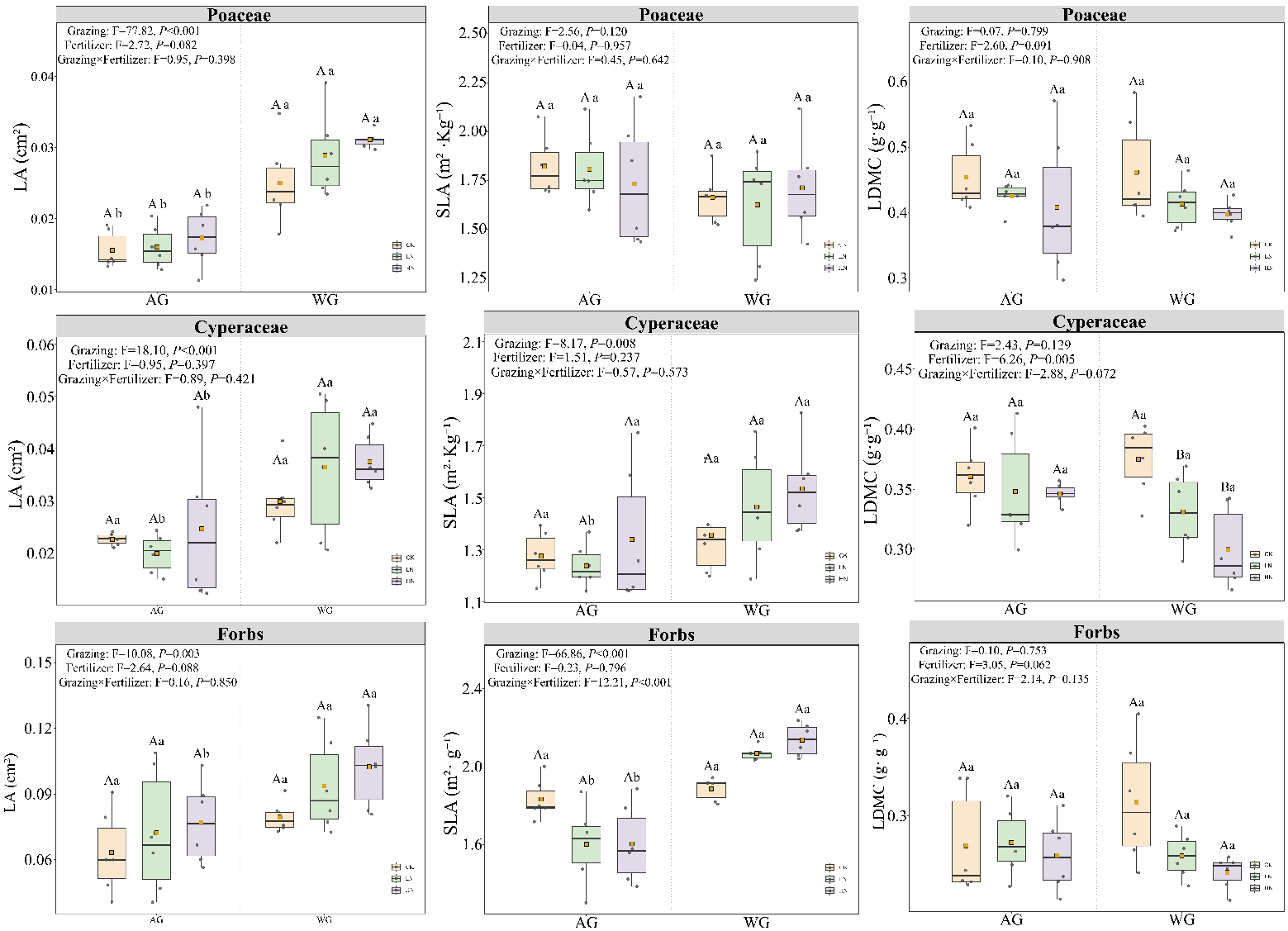
**

**Figure S1. Leaf trait responses to grazing regime and species identity across functional groups.**The figure presents leaf area (LA), specific leaf area (SLA), and leaf dry matter content (LDMC) across three functional groups (Poaceae, Cyperaceae, and Forbs) under two grazing regimes: annual grazing (AG) and seasonal winter grazing (WG). Each panel includes results from factorial ANOVA, indicating the effects of grazing, species identity, and their interaction, with corresponding F and P values. Different lowercase letters denote significant differences between grazing regimes (Tukey HSD, *P* < 0.05), while different uppercase letters indicate significant differences among species (Tukey HSD, *P* < 0.05).


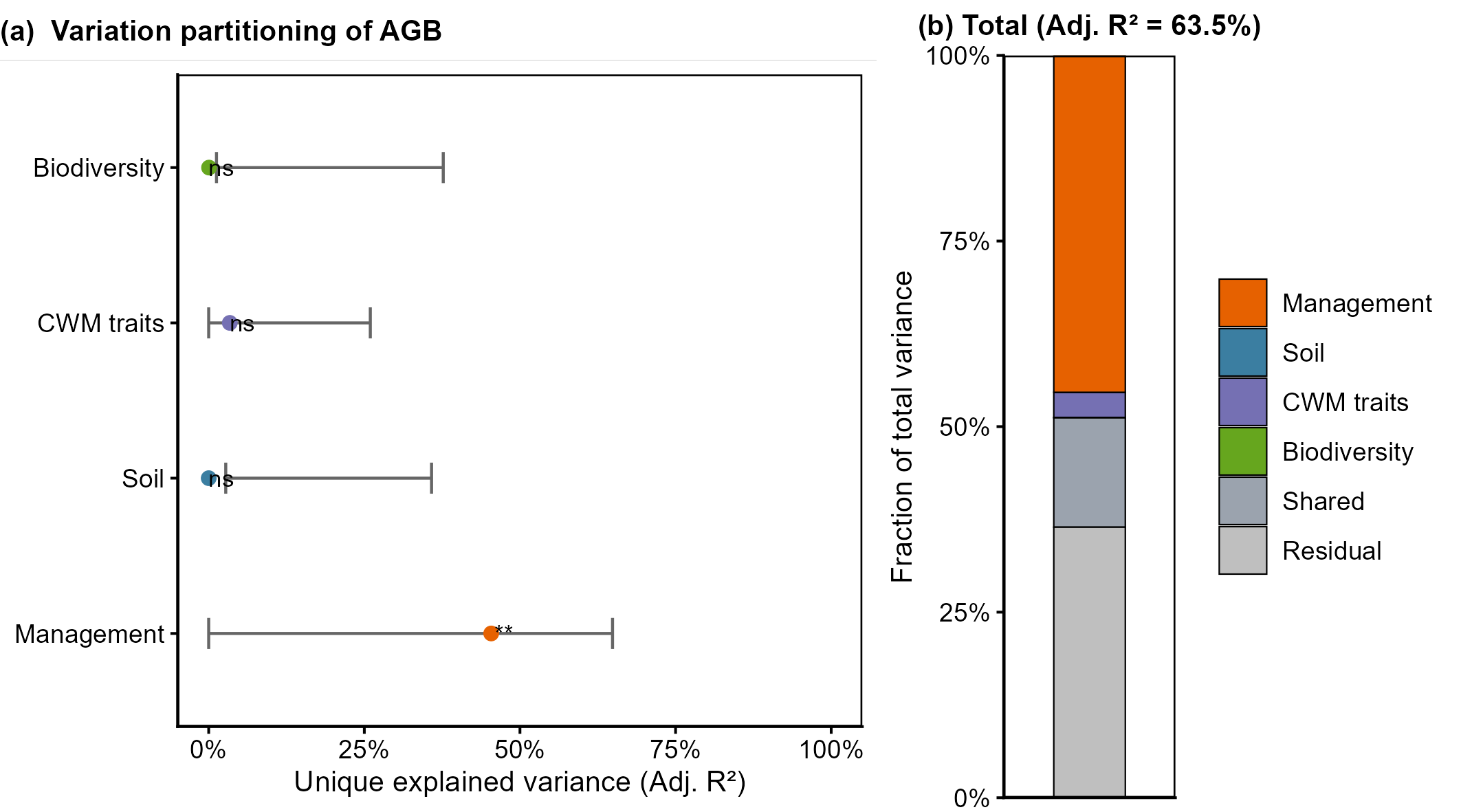


**Figure S2. Variation partitioning of aboveground biomass (AGB).** (**a**) Unique explained variance (Adj. R²) of four factor groups estimated by partial RDA/PERMANOVA, with 95% bootstrap intervals and significance levels. (**b**) Total variance partitioning (Adj. R² = 63.5%) into unique, shared, and residual components.

## Supplementary Tables

**Table S1. Type-III ANOVA and PERMANOVA: Treatment effects and effect sizes (η² / R²).**

| Test | Term | F | p | Significance | Effect size | Effect size category |
| --- | --- | --- | --- | --- | --- | --- |
| Type-III ANOVA（PC1） | Grazing (G) | 62.67 | <0.001 | *** | 0.68 | Large |
| Type-III ANOVA（PC1） | Fertilization (N) | 14.44 | <0.001 | *** | 0.49 | Large |
| Type-III ANOVA（PC1） | G×N | 4.0 | 0.029 | * | 0.21 | Large |
| Type-III ANOVA（PC2） | Grazing (G) | 6.03 | 0.020 | * | 0.17 | Large |
| Type-III ANOVA（PC2） | Fertilization (N) | 1.79 | 0.184 | ns | 0.11 | Medium |
| Type-III ANOVA（PC2） | G×N | 2.08 | 0.142 | ns | 0.12 | Medium |

Note: ns = not significant; **P*< 0.05; ** *P* < 0.01; *** *P* < 0.001. Thresholds for partial η²: <0.06 = Small, 0.06–0.14 = Medium, ≥0.14 = Large. Effect sizes for PERMANOVA are reported as R².
